# Interface Optimization and Thermal Conductivity of Cu/Diamond Composites by Spark Plasma Sintering Process

**DOI:** 10.3390/nano15010073

**Published:** 2025-01-06

**Authors:** Junfeng Zhao, Hao Su, Kai Li, Haijuan Mei, Junliang Zhang, Weiping Gong

**Affiliations:** Guangdong Provincial Key Laboratory of Electronic Functional Materials and Devices, Huizhou University, Huizhou 516001, China

**Keywords:** Cu/diamond, interface structure, thermal conductivity

## Abstract

Cu/Diamond (Cu/Dia) composites are regarded as next-generation thermal dissipation materials and hold tremendous potential for use in future high-power electronic devices. The interface structure between the Cu matrix and the diamond has a significant impact on the thermophysical properties of the composite materials. In this study, Cu/Dia composite materials were fabricated using the Spark Plasma Sintering (SPS) process. The results indicate that the agglomeration of diamond particles decreases with increasing particle size and that a uniform distribution is achieved at 200 μm. With an increase in the sintering temperature, the interface bonding is first optimized and then weakened, with the optimal sintering temperature being 900 °C. The addition of Cr to the Cu matrix leads to the formation of Cr_7_C_3_ after sintering, which enhances the relative density and bonding strength at the interface, transitioning it from a physical bond to a metallurgical bond. Optimizing the diamond particle size increased the thermal conductivity from 310 W/m K to 386 W/m K, while further optimizing the interface led to a significant increase to 516 W/m K, representing an overall improvement of approximately 66%.

## 1. Introduction

At present, with the rapid development of electronic devices towards miniaturization, high power, high integration, and efficient heat dissipation are essential to maintain high performance and prolong equipment life [1,2,3]. Every 10 °C increase in the operating temperature of an electronic device reduces its reliability by half [4]. This significantly increases the power density of electronic devices, leading to a rapid increase in heat dissipation per unit area. How to transfer the large amount of heat generated has become the key to the safe, stable, and efficient operation of electronic devices, which brings new challenges to the current electronic packaging materials and technologies [5,6]. Materials that excel in thermal conductivity are essential for enhancing the heat dissipation of electronic devices. Materials with high thermal conductivity are urgently needed [7,8]. To address these challenges, metal matrix composites that boast superior thermal conductivity and tunable thermal expansion coefficients are regarded as the most promising thermal management materials for electronic packaging applications [9,10,11]. The thermal conductivity of Cu is approximately 385–401 W/(m·K), but it has a high coefficient of thermal expansion; the thermal conductivity of diamond exceeds 2000 W/mK, but its high cost, difficult processing, and extreme brittleness limit its applications. Due to its extremely high thermal conductivity, diamond is often used as a reinforcing phase to make metal-based electronic packaging materials. The thermal expansion coefficients of Cu/Dia composites can be changed by adjusting the component ratio, with significantly improved thermal conductivity, typically ranging from 400 to 1500 W/m·K, sometimes even higher. However, the smooth and chemically inert surface of diamond, combined with its poor wettability with the Cu matrix, leads to poor interface bonding and reduced thermal performance [12,13]. Therefore, optimizing the interface structure between the diamond and Cu matrix is crucial for improving the thermal conductivity of the metal matrix composites.

For metal matrix composites, the interface region plays a decisive role in the overall performance of the material. In the process of preparing composite materials, if the interface structure between the matrix and the reinforcement phase is not well controlled, the excellent properties of the composites will be greatly reduced [14]. A large body of literature shows that if the interface quality of diamond and Cu is poor, the thermal conductivity of the Cu/Dia composites not only becomes lower than that of copper but is also much lower than that of diamond with the addition of diamond [15,16,17]. Due to the significance and complexity of the interface in Cu/Dia composite materials, the optimal design of the interface has become a research topic of great concern to scholars [18,19]. The alloying of Cu matrix and strong carbon compounds can significantly improve the infiltration and interface bonding between Cu and diamond [20,21,22]. The strong carbon compounds and diamond form an interface layer, which increases the diffusion and reaction with the diamond surface and improves the wettability of Cu and diamond [23]. This interface can also promote the coupling of phonons and diamond phonons in the Cu matrix, thereby improving the thermal conductivity of the composite. Weber [24] found that the alloying elements Cr and B in the matrix have an effect on the thermal conductivity and thermal expansion coefficient of the composite. The research indicated that as the content of alloying elements in the matrix increases, the bonding at the two-phase interface is significantly enhanced. However, upon further increasing the alloying elements, an optimal content was identified where the thermal conductivity and thermal expansion coefficient of the composite material reached their peak. Both Bai and Chu et al. found that the thermal conductivity of Cu/Dia composites first increased and then decreased with the increase in Zr and B contents, and the maximum thermal conductivity was obtained at 1037 °C and 1.2 wt.% Zr content [11,25]. Kang et al. coated diamond particles with a W layer and prepared Cu/Dia (W) composites by pressure infiltration method [26]; the results showed that the W coating played an effective sticking role and had good interface modification. In the development of Cu/Cr/diamond composites, the latest research focuses on enhancing the thermal conductivity of the materials through precise composition adjustment and innovative fabrication techniques [27,28]. Specifically, the use of Cr alloying technology to improve the performance of the copper matrix, combined with Cr-coated diamond particles, effectively optimizes the interface structure, thereby significantly enhancing the thermal conductivity [29,30]. Additionally, the introduction of chromium compound layers and the construction of a three-dimensional diamond network create an efficient pathway for phonon transport, further improving the efficiency of heat conduction. To meet the heat dissipation requirements of high-power electronic devices, research is ongoing to optimize the particle size and fabrication process of the composites and to further improve the comprehensive thermomechanical properties of the materials by enhancing the interface bonding strength. Therefore, to further investigate the influence of alloying elements on the interface characteristics of Cu/Dia composites, this study focused on analyzing the effects of different Cr contents and various diamond particle sizes on the thermal conductivity of the composites, seeking to achieve the optimal alloying element ratio for the best thermal conductivity.

In this work, the optimal method for fabricating Cu/Dia composites with high thermal conductivity was explored. The Cu/Dia composites were prepared using the SPS technique with the optimized process, and the influence of Cr content on the interfacial products and thermal properties of the Cu/Dia composites was investigated. This study will establish a theoretical framework for enhancing the thermal conductivity of Cr-modified Cu/Diamond composites through the effective design and manipulation of the interface between Cu and diamond.

## 2. Experimental

### 2.1. Raw Materials

**Figure 1:**
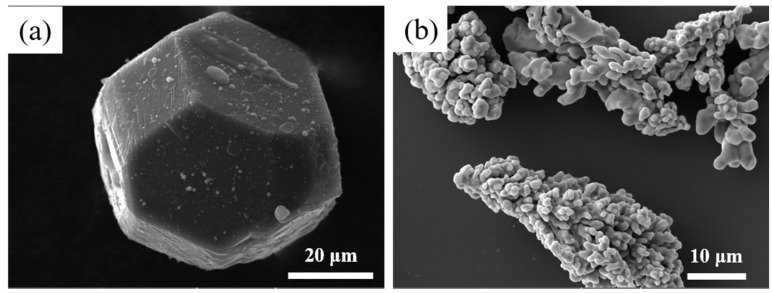
SEM micrographs of original diamond (**a**) and Cu powder (**b**).

Commercial purity Cu powder (99.99% purity) was used as a matrix material, and the Cu powder utilized was produced by Shanghai Macklin Biochemical Co., Ltd. with the product code C805729. The diamond crystal grains were purchased from Huanghe Whirl wind company. The morphology of the raw materials is shown in Figure 1.

### 2.2. Sample Preparation

The diamond particles, Cr powder, and Cu powder were weighed according to the set mass ratio and placed into a ball milling jar, followed by adding an appropriate amount of ball milling beads. The ball milling beads and the weighed powder were added to the jar in a ball-to-powder ratio of 10:1. To prevent the oxidation of Cu powder during the ball milling process, a vacuum pump was used to evacuate the air. The vacuum ball milling jar was then placed into a planetary ball mill and milled for 5 h. The grinding parameters were set as follows: rotation speed of 200 rpm, 15 min for forward rotation, and 15 min for reverse rotation with a 4 min interval. After ball milling was completed, the beads and powder were separated using a sieve. The obtained Cu/diamond powder was placed into a graphite mold, and the powder was then pressed into discs with a diameter of 13 mm at a pressure of 50 MPa and sintered under vacuum at the set temperature to form the shape. The sample preparation was carried out using a Spark Plasma Sintering (SPS) machine model SPS-12000-10AT (manufactured by Shenzhen Xingyuan Discharge Plasma Technology Co., Ltd., Shenzhen, China). During the sintering process, the sample was first heated to 500 °C at a rate of 50 °C/min; then, pressure was applied while continuing to heat to the set sintering temperature. After reaching the set temperature, it was maintained for 20 min to ensure full densification. After sintering was completed, the power was turned off and the sample was allowed to cool in the furnace. The pressure was slowly released when the temperature dropped to 200 °C. The diamond volume fraction amounted to approximately 40% of all samples. The Cu/Dia composites with this composition can effectively enhance thermal conductivity while avoiding excessive reduction in the material’s strength and toughness.

### 2.3. Characterization

The composites obtained were of a cylinder geometry with diameter of 13 mm and thickness of 5 mm. The surface morphology and distribution of elements for the Cu/Dia samples were studied by scanning electron microscope (SEM, Tescan VEGA3 XMU, Brno, Czech Republic). The composition was confirmed via energy dispersive spectroscopy (EDS) with a 30 kV accelerating voltage. The thermal conductivity (λ) of the room temperature-sintered composites was determined using the thermal diffusivity (α), density (ρ), and specific heat capacity (C_p_) values based on the following formula: λ = α × C_p_ × ρ. The thermal diffusivities of Cu/Dia composites were measured by thermal conductivity tester (LFA467HyperFlash, Netzsch, Selb, Germany). The different phases within the composite material’s structure were identified by X-ray diffraction (XRD, Rigaku miniflex600, Tokyo, Japan) using Cu Kα radiation. The Archimedes principle method was used to determine the density of the Cu/Dia composite materials with different compositions.

## 3. Results and Discussion

### 3.1. Effect of Particle Size on Agglomeration

**Figure 2:**
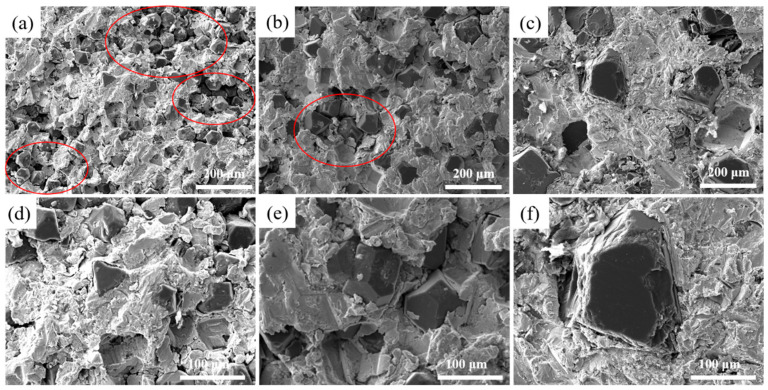
Microstructure of diamond/Al composites with different particle sizes. (**a**,**d**): 40 μm; (**b**,**e**): 80 μm; (**c**,**f**): 200 μm.

Figure 2 illustrates the microstructure of Cu/Dia composites with varying particle sizes at 900 °C. It is observed that when the diamond particle size is small, a pronounced aggregation phenomenon occurs among the particles, as indicated by the red circle in the figures a and b. As the size of the diamond particles increases, the degree of aggregation gradually diminishes. As depicted in Figure 2, smaller particle sizes are more prone to aggregation during the fabrication process. This phenomenon can be attributed to the fact that diamond particles with smaller dimensions possess a larger specific surface area and higher surface energy. The higher surface energy indicates stronger interparticle attractions, leading the particles to cluster together in an effort to reduce the overall system energy. Consequently, the formation of small pores within the aggregates and larger pores between them collectively increases the material’s porosity and results in a decrease in density. As the diamond particle size increases, the agglomeration gradually decreases, and when the particle size reaches 200 μm, the diamond particles are uniformly distributed within the Cu matrix.

**Figure 3:**
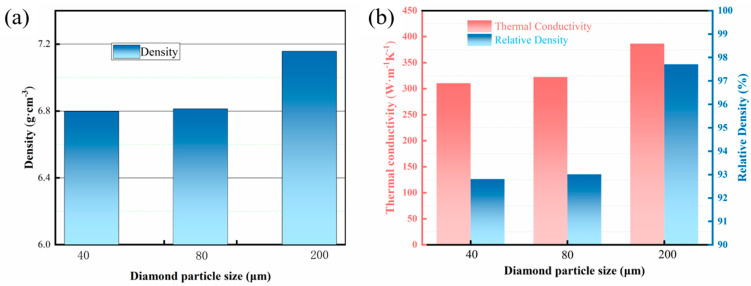
Density and thermal properties of composites with various particle sizes. (**a**): Relative density; (**b**): Thermal conductivity and actual density.

Figure 3 shows the effect of diamond particle sizes on the density and thermal properties of the composite materials at 900 °C. As the diamond particle size increases, both the relative density and actual density exhibit an upward trend. Particularly, when the diamond particle size increases from 40 μm to 200 μm, the relative density significantly increases from 92.8% to 95.01%. This indicates that larger diamond particles contribute to improving the overall density of the composite materials. With the increase in diamond particle size, the densification of the diamond/Cu composite material gradually improves. This might be because larger diamond particles are less prone to agglomeration in the composite materials, thereby reducing porosity and increasing the overall density of the materials. Agglomerated particles cannot be distributed uniformly in the matrix material, leading to non-uniform particle distribution throughout the composite materials and resulting in the formation of voids around the agglomerated areas. The agglomerated diamond particles hinder the flow of the matrix material, making it difficult for the matrix to fill the gaps between particles, thus increasing the porosity. The reduced interface bonding area and weakened bonding strength between the agglomerated particles and the matrix material also affect the overall densification of the composites. Additionally, within a given volume, as the particle size decreases, the number of interfaces increases, which in turn leads to a higher number of interfacial voids, resulting in a lower overall density.

Figure 3b presents the thermal conductivity and actual density of composites with different particle sizes. The results indicate that the thermal conductivity of the composites gradually increases with the enlargement of the diamond particle diameter. When the diamond particle diameter reaches 200 μm, the thermal conductivity reaches its highest value. The uniform distribution of particles plays a positive role in enhancing the thermal conductivity of the composites. In Cu/Dia composites, the diamond particles act as excellent thermal conductors and are uniformly distributed, which helps to construct effective thermal conduction paths and thus improves the overall thermal conductivity. Conversely, particle agglomeration or non-uniform distribution can easily form defects within the material, such as voids and cracks, which may become areas of stress concentration and consequently weaken the material’s performance. Uniformly distributed particles help to reduce these internal defects and optimize interface characteristics. The improvement in relative density can enhance the efficiency of heat transfer between particles, thereby enhancing the overall performance of the composites.

The standard theoretical models employed for predicting thermal conductivity are the Maxwell–Eucken model and the Discrete Element Method (DEM) model [31,32]. The prediction formula for the Maxwell–Eucken model is presented in Equation (1), while the DEM model’s prediction formula is detailed in Equation (2) [31]:

(1) $$\lambda =\frac{\lambda_{Cu} [2\lambda_{Cu}+\lambda_{diamond}+2(\lambda_{diamod}-\lambda_{Cu})V_{\mathrm{diamond}}]}{2\lambda_{Cu}+\lambda_{diamond}-(\lambda_{diamond}-\lambda_{Cu})V_{diamond}}$$

(2) $$\sqrt[{3}]{\frac{\lambda }{\lambda_{Cu}}}\left(1-V_{diamond}\right)=\frac{\frac{\lambda_{diamond}}{\lambda_{Cu}}-\frac{\lambda }{\lambda_{Cu}}}{\frac{\lambda_{diamond}}{\lambda_{Cu}}-1}$$

In these equations, *λ* represents the thermal conductivity of the composite, *λ_Cu_* denotes the thermal conductivity of the Cu matrix, *λ_diamond_* refers to the theoretical thermal conductivity of diamond, and *V_diamond_* is the volume fraction of diamond within the composite.

The thermal conductivity of the composites is influenced by the particle size of diamond, as it impacts the interface thermal resistance [33,34]. The modified effective thermal conductivity, *λ’d_iamond_*, is utilized in place of the original *λ_diamond_*.

(3) $$\lambda \prime =\frac{\lambda_{diamond}}{1+\frac{\lambda_{diamond}}{rh_{c}}}$$

In the above equation, r denotes the size of the diamond particle, and *h_c_* represents the thermal conductivity at the interface between the Cu matrix and the diamond particles. As the diamond particle size decreases, the interface area increases, which diminishes phonon scattering and enhances phonon-mediated heat transfer. Consequently, when the volume fraction of diamond remains constant, a reduction in particle size leads to a decrease in the thermal conductivity. Conversely, as the size of the diamond particles increases and the interfacial gap diminishes, the thermal conductivity of the composite progressively improves.

### 3.2. Effect of Different Sintering Temperatures on the Interface

**Figure 4:**
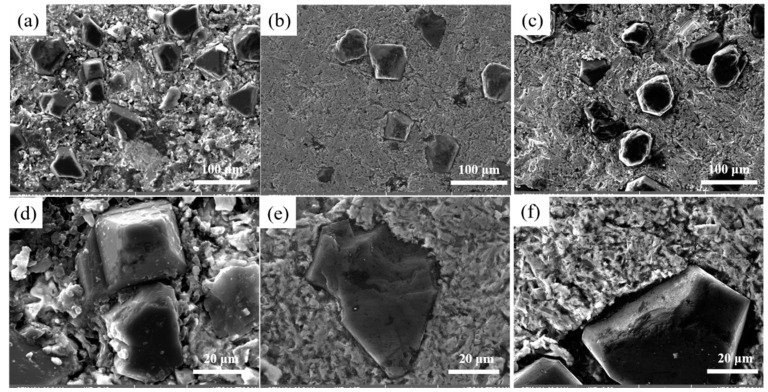
The impact of different sintering temperatures on the interface structure. (**a**,**d**) 800; (**b**,**e**) 900; (**c**,**f**) 1000 °C.

Figure 4 shows the effect of different sintering temperatures on the interface structure of Cu/Dia composites. As the temperature increases, the interface bonding condition improves under a certain pressure. However, when the temperature reaches 1000 °C, noticeable gaps appear at the interface. At an appropriate sintering temperature, Cu and diamond can form good chemical bonds, thereby enhancing the interface bonding strength. If the sintering temperature is too low, the interface reaction will be insufficient, leading to weaker bonding forces. Conversely, if the sintering temperature is too high, due to the difference in thermal expansion coefficients between Cu and diamond, a significant expansion discrepancy will occur at high temperatures, resulting in the formation of tiny gaps at the interface upon cooling. This reduces the wettability of Cu on diamond and subsequently affects the interface bonding effectiveness.

### 3.3. The Impact of Different Cr Contents on the Interface

**Figure 5:**
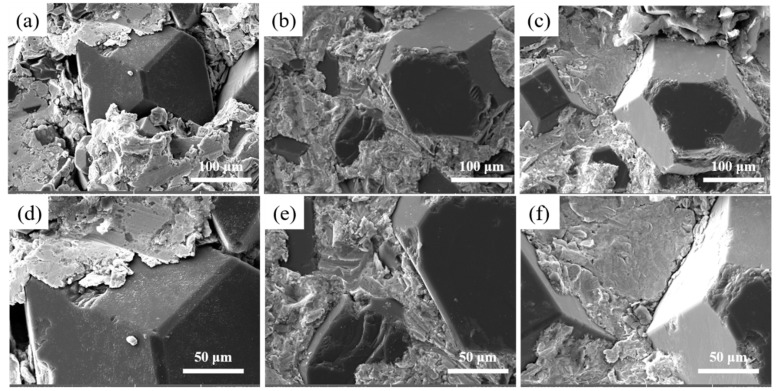
The influence of different Cr contents on the interface. (**a**,**d**) 0; (**b**,**e**) 1%; (**c**,**f**) 3%.

**Figure 6:**
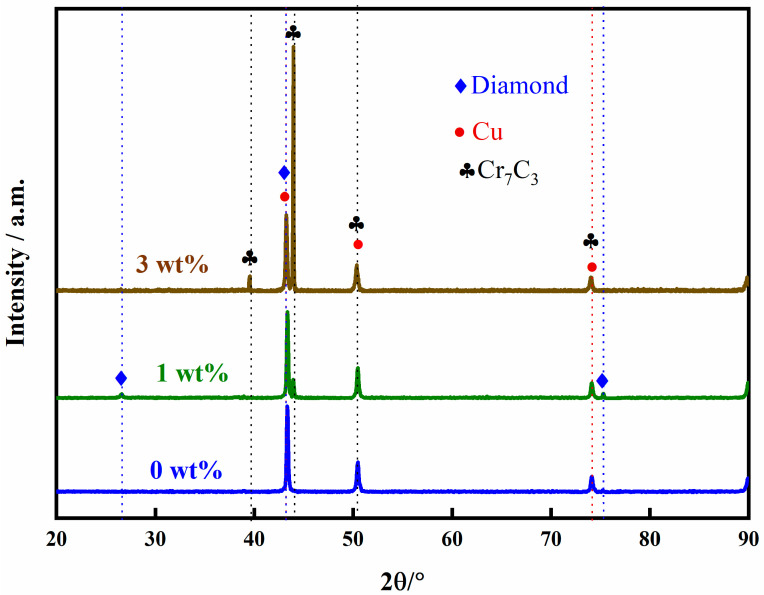
XRD of composite materials with different Cr contents.

Figure 5 illustrates the effect of different Cr contents on the interface structure of Cu/Dia composites at 900 °C. Figure 6 provides the XRD analysis of composites with various Cr contents. These composites consist of three distinct regions: the diamond reinforcement phase, the interface layer, and the Cu matrix, with the diamond reinforcement phase connected to the Cu matrix through the interface layer. At the interface without Cr, the bonding force between the diamond particles and pure Cu is weak, as shown in Figure 5a,d. It is clearly evident from the figures that despite the application of a certain pressure during manufacturing, there is a noticeable gap between the two phases due to the poor wettability between diamond and Cu, resulting in a lower thermal conductivity of the Cu/Dia composites [35]. Figure 5b,e shows the image of a Cu/Dia composite containing 1 wt% Cr; the bonding between diamond and Cu is significantly stronger than that in the previous interface. With the increase in Cr content in the Cu alloy, a transition from weak to strong interface bonding can be observed in the Cu/Dia composites. A small amount of Cr in the matrix is difficult to form a uniform interface due to insufficient chemical reaction. When the Cr content is increased to a sufficient value (3%), it can be confirmed that a strong interface bond is formed between the diamond particles and the Cu matrix, as shown in Figure 5c,f.

From Figure 6, the XRD of composites with different Cr contents reveals that the interface reaction product is Cr_7_C_3_. At the interface, the Cr element has undergone a chemical reaction with the diamond, leading to an improvement of the interface. It can be observed that as the Cr content increases from 0% to 3%, the constituent phases at the interface become tightly bonded, indicating that the interfacial carbide layer can act as a “binder” to firmly adhere the diamond filler to the Cu matrix. The Cr element reacts chemically with the diamond to form a layer of chromium carbide, which serves as a transition layer, enhancing the chemical bonding force between Cu and diamond. Extensive literature research indicates that Cu and diamond have poor wettability [22,36]; the addition of Cr promotes the diffusion of Cu atoms on the diamond surface, and this diffusion of Cu atoms on the diamond surface aids in the formation of a stronger interfacial bond.

**Figure 7:**
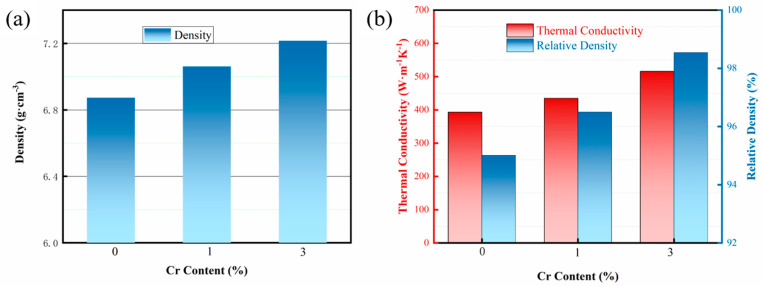
Density and thermal properties of composites with various Cr contents. (**a**) Density; (**b**) Thermal conductivity and Relative density.

Figure 7 presents the density and thermal properties of composites with varying Cr contents. When Cu and diamond are in direct contact, the presence of numerous voids at the interface results in a thermal conductivity of only 393 W/m K. As the interface layer is optimized, both the density and compactness of the composites increase, leading to a rising trend in the thermal conductivity coefficient. When the Cr content reaches 3%, the thermal conductivity attains its maximum value of 516 W/m K. The relationship between thermal conductivity and relative density indicates that as the interfacial voids decrease, both the thermal conductivity and relative density of the Cu/Dia composites gradually improve. This is because samples with lower relative density contain a higher volume of pores, and the thermal conductivity of gases is very poor, at just 0.0267 W/m K, which is negligible compared to the Cu matrix and diamond. Moreover, as the relative density decreases, the phonon scattering intensifies, reducing the mean free path of phonons and thus decreasing the thermal conductivity of the composites. This experimental result also fully demonstrates the significant role that relative density plays in the thermal conductivity of composite materials.

When calculating the thermal conductivity of composite materials using a thermal conductivity prediction model, the interfacial thermal conductivity h_c_ between Cu and diamond is introduced, and its expression is as follows:

(4) $$h_{c}=\frac{1}{4}\rho_{Cu}c_{Cu}C_{Cu}\eta_{Interface}$$

In the formula, *ρ_Cu_* stands for the density of Cu, *c_Cu_* is the specific heat capacity of copper, *C_Cu_* indicates the phonon propagation speed in Cu, and *η_Interface_* represents the phonon heat dissipation coefficient at the interface of Cu and diamond. This coefficient can be calculated using the phonon mismatch model, as specified in Equation (5):

(5) $$\eta_{Interface}=\frac{2Z_{Cu}Z_{diamond}}{{\left(Z_{Cu}+Z_{\mathrm{diamond}}\right)}^{2}}\frac{C_{\mathrm{DCu}}}{C_{Ddiamond}}$$

*Z_Cu_* and *Z_diamond_* are the respective phonon impedances of the Cu matrix and the diamond (Z = ρC_D_, C_D_ is the Debye sound velocity). *C_Ddiamond_* denotes the phonon velocity of diamond. The phonon velocities for both the Cu matrix and the diamond are determined using Equation (6):

(6) $$C_{D}=\frac{1}{\sqrt{\frac{1}{2}\left(\frac{1}{C_{1}^{2}}+\frac{1}{C_{t}^{2}}\right)}}$$

*C*_1_ represents the phonon velocity traveling through the matrix in the longitudinal direction, while C_t_ is the phonon velocity propagating laterally within the matrix. The heat transfer in the Cu/Dia composites primarily relies on the phonons generated in the diamond. The interface and the pores are the key factors that influence thermal conduction in these composites [23]. The relationship between the thermal conductivity of Cu/Dia composite materials and the thickness of the interfacial layer can be explained by the following reasons: The absence or minimal addition of Cr content results in direct contact between the diamond and Cu, forming a discontinuous interface. Defects or voids at the interface increase the interfacial thermal resistance, significantly reducing the thermal conductivity of the Cu/Dia composites. The thermal conductivity improves with the optimization of the interface. Without the intervention of Cr alloy compounds at the interface, there are numerous voids present. As the Cr alloy compounds continuously distribute within the interfacial layer, the thermal conductivity of the Cu/Dia composites begins to show an upward trend. This indicates that interfacial modification of the composites through Cr alloying can introduce an interfacial layer between the Cu matrix and the diamond reinforcement phase, thereby improving the interfacial bonding and enhancing the thermal conductivity of the Cu/Dia composites. The role of the interfacial layer is mainly reflected in two aspects: first, it increases the wettability between the Cu matrix and the diamond, improving the interfacial quality of the composites and significantly reducing the interfacial thermal resistance; second, the acoustic properties of the Cr compounds are intermediate between those of Cu and diamond, which can increase the efficiency of phonon transmission, thus effectively enhancing the interfacial thermal conductivity. However, as the Cr content at the interface continues to increase, the thermal conductivity will exhibit a decreasing trend. On one hand, a certain amount of Cr_7_C_3_ is formed at the interface between the diamond and Cu, which improves the wettability and interfacial bonding strength between the matrix and the diamond, allowing the Cu/Dia composites to fully utilize the high thermal conductivity advantage of diamond. On the other hand, the thermal conductivity of Cr_7_C_3_ is lower than that of Cu and diamond, with only 19.1 W/m K. An excessive content of Cr_7_C_3_ will increase the interfacial thermal resistance, thereby reducing the thermal conductivity of the composites [37]. Therefore, strict control of interfacial products is necessary, as they are influenced by temperature and time. Future work will focus on optimizing the Cr diffusion layer thickness to boost the thermal conductivity of composites.

Compared with those reported in the literature for similar Cu/Dia composites, the results showed that after optimizing the interface structure, the thermal conductivity of our composites reached 516 W/mK, which is lower than some of the highest values reported (810 W/mK [38] and 696 W/mK [39]). This may be due to factors such as the lower diamond content, interface quality, particle size, and sintering temperature. Although our results are slightly below some of the highest values reported in the literature, the outcome remains competitive given the lower diamond content and reduced sintering temperature of our composites. Furthermore, our research, which focuses on enhancing thermal conductivity through interface optimization, offers valuable insights for the development of Cu/Dia composites with superior thermal performance.

## 4. Conclusions

Cu/Dia composites with Cr addition were fabricated using the SPS technology. The SPS process was refined, and the interfacial structure and thermal conductivity were assessed. Key findings are summarized below:

1. As the diamond particle size increases, the agglomeration of the composites gradually diminishes, and when the diamond particle size reaches 200 μm, the diamonds are uniformly distributed within the matrix. With the increase in sintering temperature, the interface bonding is optimized initially and then weakened, with the optimal sintering temperature being 900 °C.
2. The addition of Cr elements to the Cu matrix leads to the formation of the Cr_7_C_3_ phase after sintering, which increases the relative density of the composite material and enhances the bonding strength between the diamond and the matrix, transitioning the interface from a physical bond to a metallurgical bond.
3. With the optimization of diamond particle size, the thermal conductivity of the composites increased from 310 to 386 W/m K; after interface optimization, the thermal conductivity further rose to 516 W/m K, with an increase of approximately 66%.

## Data Availability

The original contributions presented in this study are included in the article. Further inquiries can be directed to the corresponding authors.
